# Book Reviews

**Published:** 1818-08

**Authors:** 


### CRITICAL ANALYSIS
### *OF RECENT PUBLICATIONS,*
IN THE
### DIFFERENT BRANCHES OF PHYSIC, SURGERY, &c.

*Surgical Observations; being a Quarterly Report of Cases in Surgery; treated in the Middlesex Hospital, in the Cancer Establishment, and in private Practice. Embracing an Account of the Anatomical and Pathological Researches in the School of Windmill Street.* By CHARLES BELL. Vol. II. Longman and Co.

*(Continued from p. 62.)*

WE left Mr. Bell, in our last Number, at the close of his philippic against the army-surgeons, for asserting that syphilis was curable without mercury, which, it will be remembered, contains some very singular specimens of the reasoning faculty of the author; we have now his experience detailed on the subject of *Some Affections of the Urethra and Bladder consequent to Injuries and Obstructions from Calculi.*

This report seems principally intended for his pupils, to whom information of any kind or degree may be acceptable; but we cannot help remarking, that even to them it is necessary to be concise, intelligible, and accurate; instead of which, we have a very small quantity of matter, spun out into great space,—language employed, that we defy even the author himself to understand; and so mixed up with bad English, and worse Latin, as to be discreditable to a school-boy. Of what importance is it in a work of a didactic nature, to let us know that a patient is "now in his natural character of a great good-natured fellow of thirty, that would run his round black head into any kind of mischief to shew his gratitude?" After his patient dies, "now, unfortunately, he can understand the case;" though, in fact, the misfortune is not that he understood it then, but mistook it before: directs his leeches to be applied ad im*o* abdomin*e!*, and gives his medicines ter in *diem!*

The cases severally follow with their titles: The first is denominated *Strange Misapprehension in a Case of Rupture of the Urethra.*—The patient had been long troubled with a stricture in the urethra near the bladder; four days before the accident, was unable to make water, and, in attempting to introduce the catheter, ruptured the urethra. The scrotum in the course of a night had enlarged to an extraordinary size; the patient was speechless; his eyes fixed, and his features working in convulsions; he could no longer swallow: the bladder was observed to have risen above the pubis; the scrotum was full of urine, and there was a hard tumour in the perineum. A very sapient practitioner, and, doubtless, a man of *taste*, had been previously called to him; had introduced the catheter; sucked the bladder—drew no urine into his mouth, but oil! The bladder could not be felt in the rectum, therefore could not be punctured, and the patient died.

*Tumour in the Perineum and Extravasation of Urine from Stones obstructing the Urethra.*—The patient had passed many calculi at different times; the scrotum and penis were now found distended; his voice was small; hand cold, and pulse feeble.

The flexible catheter could not, at first, be passed into the bladder, but found its way in a sac formed by suppuration, which contained urine. At length, it passed through the abscess, and reached the bladder, by which a pint and a half of fetid urine was drawn off, and a stone was felt: punctures were made on each side of the septum scroti, from which the fluid drained copiously; mortification began the day after his admission, and on the fourth he died. The scrotum had much putrid matter in it; a cavity was seen in the perineum filled with small stones: in the prostate were also many calculi; the bladder was contracted, and the inner coat inflamed.

*Common Case of Rupture of the Urethra.*—A man who had stricture without knowing it, ruptured the urethra: the urine was extravasated into the scrotum, penis, and lower part of the belly. An incision into the lower part of the scrotum effectually drained off the fluid, but the integuments of the pubes suppurated.

*Rupture of the Urethra from a Fall.*—A man fell astride on the ballustrade of the staircase, since which he could not pass his urine. The bulb of the urethra, and the crura penis, were much swoln: nine leeches were applied, a laxative was given, and he was placed in the tepid bath. The next day the catheter could not be introduced until the swelling of the perineum was opened and a quantity of blood let loose. The silver catheter being retained, the wound suppurated and closed, but broke out again, discharged matter and urine, and became fistulous; instead of keeping the catheter in the bladder, a hollow bougie was introduced three times a-day.

*Wound in the Perineum.*—A wheel went over the top of the thigh and the left haunch-bone; the patient bled much from the fundament, and his fæces have passed from him involuntarily with blood. He was bled and purged; the catheter in the hands of the house-surgeon seemed to pass, but Mr. Bell failed in the attempt to introduce it; and it appeared, that, in the former essay, it had passed out of the urethra into the wound, and did not reach the bottom. The wound is irregular and lacerated; the ramus of the ischium appeared fractured. After introducing the finger, on withdrawing it, blood and urine escaped, with a smell of fæces.

Eight leeches were applied to the abdomen, and a saline mixture with opium ordered; but, as this concise direction may not be enough for the literati, we give it in Mr. Bell's *own* Latin:—App. Hirudines octo *ad imo abdomine* et Capiat Misturæ Salinæ Cochlea*rea* tria ampla, cum Tinct. Opii, gtts. xv. *ter in diem.*

On a consultation respecting the case, it appeared that the tumour arose above the pubes, but not quite to the umbilicus; the general swelling of the belly was increased, but the tumour of the bladder was not enlarged, nor could the bladder be felt in the rectum. The urine drained off freely, so that puncturing the bladder was not resorted to.

After death, it appeared that the tumour was caused by the turns of the colon, which had attached themselves to the inflamed bladder.

*Abscess of the Perineum from Diastasis of the Ossa Pubis.*—No stricture existed to obstruct the urine; the man in striding over a sack of tallow to lift it up, felt something give way at the lower part of the belly; he became lame, and then followed the inflammation and suppuration, and ultimately died of a dysenteric affection. On dissection, the cause of death was manifest: the symphisis pubis was found destroyed, and the ossa pubis near their union carious, and surrounded by matter; on each side, a portion of the bone was dead and exfoliating; a strong preternatural ligament connected the bones loosely at the upper part.

*Case requiring the Bladder of Urine to be punctured.*—The patient was under great distress from obstruction of urine; had strictures many years, and suffered much from temporary obstructions; was formerly under Mr. Bell's care for the same complaint, when a bougie was introduced into the stricture; he was then made to strain, at the same time the bougie was withdrawn, and a little urine flowed. For the last fourty-four hours of the present attack not a drop of water had passed; he was in great danger; had been bled and put into the warm bath with opiate clysters: neither the wax or catgut bougie could be introduced into the stricture; he was lethargic, and delirium soon to be expected.

The bladder being much distended and tense, rising to the umbilicus, was punctured through the rectum,* and four pints of urine drawn off to the inexpressible relief of the patient, who, notwithstanding, did not survive more than nine days, though the urine escaped both through the rectum and by the urethra. An examination of the body was not allowed, but the bladder was taken from the lower opening of the pelvis. The coats were thickened, and the inner surface was studded with white spots of coagulable lymph, which are a consequence of stricture, and independent of the operation; a bloody and ropy fluid was contained in the bladder; the prostate was surrounded by abscesses, from which thick white pus issued, the urethra largely ulcerated so as to be rendered quite irregular; and the ulcerations had a hardened base, indicating that they had been of some standing.

*Bursting of the Urethra and Sloughing of the Perineum, where it was necessary to Puncture the Bladder.*—About the middle of March, in this case, was seen a small very hard tumour attached to the lower part of the urethra, and attended with discharge from the canal. Although a bougie indicated that the urethra was obstructed and compressed by the swelling, he could make urine freely: the patient was ordered to live low, to rest on the sofa, to apply leeches, and to foment the part.

In five days time, he became much worse; he made water with difficulty, and complained of spasms at the neck of the bladder: leeches were applied to the verge of the anus; he took mucilaginous tepid drink, an anodyne with the liquor potassæ, and had a starch clyster, from which he obtained relief.

In about eight days time, he became again worse; the tumour or swelling occupied the whole root of the penis, and the prepuce was œdematous; he had shivering, was feverish and exhausted; the bladder had risen above the pubes, but the urine still flowed so as to deceive him. Mr. Bell drew off the urine by a small catheter, ordered him a dose of calomel and opium and a fomentation to the perineum; and, as it appeared evident that the inflammatory tumour and abscess, which had formed by the side of the urethra, received the urine, keeping up the irritation and inflammation, the abscess was opened.

Two days after, it was impossible to introduce the catheter, from the extensive inflammation round the original obstruction, and the irregularity of the opening by ulceration into the urethra; the abscess was, therefore, opened more freely, when a gum catheter was passed directly through it into the bladder, by which a great quantity of offensive matter and urine was let off.

After this there was an interval of some weeks, during which he went on improving under the care of his apothecary, when Mr. B. was again called; though he spoke of his comparative ease, yet being thinner and much reduced, with a frequent pulse and dry tongue, and the catheter which had been retained in the bladder being suspected to be a cause of irritation, it was withdrawn, and, instead of being suffered to remain in, was introduced night and morning; this plan was, however, soon abandoned, from the impossibility sometimes of introducing it.

About a fortnight after, symptoms of that morbid state of mind came on which sometimes attend disease of the bladder; in an irritable fit, he drew out the catheter and threw it to the end of the room, which could not afterwards be introduced; the urine was completely obstructed, and the bladder so distended upwards to the navel as to render its puncturing necessary, which was done by passing a trochar above the pubis; this operation succeeded perfectly, but the powers of life were gradually exhausted, and death closed the scene on the 22d of June.

*Another complicated Case, where, instead of Puncturing the Bladder, the Urethra was opened.*—The subject of this narrative, while under the care of other surgeons, had for the last seven years been liable to frequent and alarming attacks of retention of urine; for three years "has never made a stream of urine, but has had constant stillicidium;" *he* could introduce a large-sized bougie nine inches in the urethra, though still he did not make his water better; but, as on passing a small bougie slightly curved, it was obstructed at six inches, and firmly held,—proving that it was a stricture, not a lacuna, it was evident the passage, nine inches in depth, was a false one. By attention to this stricture and his general health, he recovered in two months the power of making water, and his bladder was more retentive, but his great distress was a complaint in the lower part of the rectum.

On the ninth of October, after some excess, his urine became obstructed, for which anodyne clysters, a bath, and bleeding, were ordered, but the latter he would not submit to: by these means, and the use of the bougie, he voided a considerable quantity of mucus and urine. Next day he was very restless, but the urine flowed, and the spasms were relieved by repeated doses of an antimonial mixture.

Mr. Bell saw him after this: his urine was passed freely, mixed with mucus; his bowels in a distressing state; his stools white, and he has pain and tenderness of the belly, to which he had twelve leeches applied. The three following days his great distress was tenesmus and the passing of glairy mucus from the rectum, which increased; and at length the retention returned with fullness in the perineum: leeches were applied, and he was put into the warm bath. After this, he complained of scalding in making water, and his penis was slightly distended; when Mr. Bell was again sent for in the afternoon of this day, "he had further calls to make water, but not a drop escaped." Mr. Bell introduced a catheter down to the stricture, made an incision into the perineum at the extremity of the catheter, and, when he had pierced the fascia, a jet of pure urine followed; he then cut into the urethra, took a common trochar and pushed it slowly backwards, so as to pierce the stricture: the result of the operation was a free exit for the urine, but at length he died. On dissection, an abscess was found between the bladder and rectum, and communicating with the latter; the internal part of the prostate gland was destroyed by suppuration, its walls forming the sac of a large abscess; the rectum was ulcerated; the inside of the bladder was not inflamed, but had formed several pouches, which were full of the same ropy matter which came from the penis; the rectum was extensively ulcerated, and about four inches up the coats had a scirrhous hardness, and a large hole communicating with the abscess.

In the remarks on this case, Mr. Bell suggests, that this mode of puncturing the stricture, which he has frequently adopted, deserves more consideration than it has hitherto obtained, inasmuch as it not only relieves the bladder as effectually as a direct puncture into it, but lays the foundation of a radical cure. He condemns also the practice of some surgeons, as Dease, who use force to introduce a catheter *through* a stricture, actually breaking it down. On this point, as on many others, truth lies in the middle path: we have seen many cases where this seeming violence has been very effectual, but, doubtless, others occur in which it would be improper.

There are some cases of this disease, Mr. Bell observes, in which there is a harassing obstruction wearing the patient out: although the bladder is not distended, the incessant stimulus and irritation are attended with fever and exhaustion, with such continued torture and incessant call to pass urine day and night, that the fever terminates in delirium, in effusion of the brain, or in inflammation of the lungs; nay, even without these symptoms, the patient dies from exhaustion. In this case, the bladder cannot be punctured, as it may contain but an ounce of urine; the stricture must be cut upon, and a passage opened into the urethra behind it; by relieving the bladder from the necessity of violent and frequent action, the cause of the thickened coats is removed, they relax and allow a larger quantity of urine to collect.

The next report is to show that "*Errors in Digestion and the Irritation of Matter in the Intestinal Canal produce Pains in the Bladder and Urethra often mistaken for the Symptoms of Stricture, and for Stone in the Bladder.*—The facts contained in this report are important, inasmuch as they are not known to some of the less-experienced members of our profession, and an ignorance of them leads to serious inconvenience, and a disappointment both to the surgeon and the patient. We have seen many cases where patients have long been disquieted under apprehensions of stone in the bladder, which a few doses of magnesia or potash have effectually removed; and, as Mr. Bell observes, when the case is mistaken for stricture, the patient is subjected to the discipline of a bougie, always inconvenient, and, in the hands of an ignoramus, who alone can think of using it in such a case, often dangerous, by the production of false passages in the urethra.

A gentleman complained of pain and irritation at the neck of the bladder, and discharge from the urethra, heat in making urine, and a frequent inclination. Bougies, which he had been in the habit of using, seemed to be intercepted at the neck of the bladder: on introducing a bougie, it became entangled near the neck, and on pushing it onwards gave pain; but a larger bougie, with the point upwards, passed into the bladder, starting over some obstruction—certainly not stricture, as it was not grasped by it: it seemed to have been caused by injury done to the membrane at that part by bougies improperly used.—The patient recovered by attention to the bowels, and discontinuing the customary irritation of the bougie.

Another had excessive tenderness in the perineum, so that as he walked across the room he was afraid to bring his thighs together, could not mount his horse, or sit in his carriage, without having a hole to correspond to the perineum; a bougie passed readily, and pressure on the part produced no pain, though, when he rose to walk, his progression was as before remarked, betraying the utmost anxiety that not even the clothes should touch the perineum.—This man was cured in the same manner as the preceding.

A professional man had great pain in the bladder, accompanied with frequent desire to make water, and an increase of pain on voiding it, which was especially severe when the bladder was empty, as in cases of stone; he suffered indescribable irritation over his whole body, beginning at the lower part of the belly. These symptoms usually arose when he lay down in bed, probably owing either to the change of posture, or the influence of cold sheets, or the circumstance of his bladder being just emptied. Being urged to consider whether something in his diet had not produced the mischief, his suspicion fell on a preposterous indulgence in figs and Spanish white wine for several nights.—Purgatives, and avoiding indigestible matters, cured the complaint.

Sometimes these symptoms are occasioned by scybalæ in the rectum.

The remaining subjects are on White Swellings; the Axis of the Pelvis; on Hernia and the Operation of Lithotomy, which we reserve for our next.

---

*Observations on Dr. Wilson's Tincture, the Eau Medicinale, and other pretended Specifics for Gout;* by Dr. Williams, Ipswich. 4to. pp. 25. London, Callow, 1818.

Quackery is at all times detestable to a professional mind, more especially when it is coupled with circumstances of fraud and imposture. Our older readers cannot have forgotten, that in the pages of this Journal, several years back, an account was given of the valuable discoveries of a practitioner in London, on the subject of Gout, and the properties of the Colchicum in its treatment. Mr. Want, the gentleman in question, in giving the particulars of this remedy, distinctly stated, that, although it unquestionably possessed the power of removing the paroxysm, it was a drug of a deleterious nature, not to be trusted in the hands of the public; and gave an instance of its dangerous properties, where it produced a metastasis of gout to the abdominal viscera, which nearly cost the patient his life. As during the progress of the enquiry on this subject, the experiments which were making were not kept secret, the fact of this drug being the basis of the Eau Medicinale was known to many, among others, of the members of the Westminster Medical Society; and one person, the said Dr. Wilson, advertised and sold the tincture *as the Eau Medicinale*, and in the year 1813 published several cases, demonstrating its efficacy, as he conceived. Unfortunately, however, for this disinterested gentleman, an account of its preparation was soon after published, the bad properties which it possesses not being disguised. It was now no longer convenient to the Doctor to use the Eau Medicinale as such, and accordingly he ushers it into the world as a very great improvement on the previous medicine, under the name of Wilson's Tincture; and, the more effectually to carry on the deception, joins the discocoverer, so far as he condemns the Colchicum, and takes care to let the public know every thing that is bad respecting it, with much more than it really deserves.

The immediate origin of Dr. Williams's Tract seems to have been a statement made by Mr. Want, in one of the Monthly-Magazine Report of Diseases, to the following effect.

"It is painful to record an instance of death from the imprudent administration of Colchicum in a case of rheumatism occurring in one of our London Hospitals: too large a dose had been given through the carelessness of the nurse, which produced constant vomiting and purging, with agonizing pain in the stomach and bowels, for the space of 24 hours, until the patient was relieved of his sufferings by death. I cannot too often caution the public against this potent and insidious drug, and feel the more regret, as I was the first to make known its powers in the removal of the paroxysm of gout; though the readers of the Monthly Magazine must ever bear in mind, I have stated in the most distinct terms, it was a medicine not to be trusted in the hands of the public. The tinctures sold by Wilson, Reynolds, Hyden, and others, are prepared according to my prescription for making the French medicine, and sooner or later bring innumerable evils on the credulous patient."

Dr. Williams very naturally conceives this to be tolerable authority, writes a letter on the subject to the Suffolk Chronicle, to which Dr. Wilson responds, giving throughout the whole epistle a practical illustration of the old proverb, that some persons should have good memories. In this letter Dr. Wilson affirms that *he* discovered the composition of the Eau Medicinale, and used it in the year 1811; but that a *few months after* he abandoned it, on account of the various ill consequences that have been stated to result from its use. Now, not earlier than the middle or latter part of the year 1813 (one of his cases was dated April 1813), he published his eulogy of the Eau Medicinale, although he had abandoned the use of it in the year 1811! In the same paragraph he avers that he submitted his medicine to the different professors of the Edinburgh School, whom he named. Dr. Wilson little thought of the possibility of an enquiry being made into the truth of his statement. Dr. Williams, however sent to the professors, not one of whom had ever heard *of him* or *his medicine*. The author observes,

"It is conceded by Dr. Wilson, with what view the public will judge, that two of these 'popular medicines' are clearly mischievous: he avows that he 'discovered the true nature and composition of the *Eau Medicinale*, as early as August 1811;' that he discovered the tincture 'about the same time,' and congratulates himself on the 'immense advantages arising from its superior efficacy.' To what circumstance then shall we attribute the doctor having published in May 1813, that the *Medicinal Water* continued to support its high character of extraordinary efficacy, in regular paroxysms of gout and rheumatism,'* and that he 'plumed himself' on being instrumental in diffusing the benefits of the Medicinal Water, or Eau Medicinale, at a cheap rate in 1813; when not one word was offered in favour of the tincture, although he had ascertained its *superior efficacy* two years previously? *At what time* did Dr. Wilson announce to the public the discovery of his Tincture? *At the very period* when, by a general conviction of the pernicious effects of the Eau Medicinale, its sale, by *that name,* became comparatively nothing. *Immediately* Dr. Wilson had recourse to the ingenious stratagem of discarding the Medicinal Water, and strenuously recommending a Tincture, which is a similar preparation, and *bonâ fide* the Eau Medicinale. If the Tincture and the Eau Medicinale were discovered by him at the same time, as Dr. Wilson declares, and he then considered the effects of the latter as pernicious or fatal, why did he delay to announce the Tincture? or why did it remain unknown till the Eau Medicinale became unpopular and almost unsaleable? The obvious solution of this mystery is, that the Tincture, in substance and in truth, is the Eau Medicinale; which at once removes the difficulty and inconsistency of the Medicinal water having been strongly recommended by Dr. Wilson in 1813, though the '*immense advantages*' of the Tincture were discovered, by his own acknowledgment, in 1811!—but I shall enter more fully upon this subject hereafter. Dr. Wilson next asserts, that he submitted the results of his experiments, in the course of the ensuing year, to the professors of botany, medicine, and therapeutics, in the University of Edinburgh. What conclusions shall be drawn from this assertion, after a perusal of the following letter, which was transmitted to me, from an eminent physician at Edinburgh, by a gentleman, to whom I wrote upon the subject, observing that I supposed Dr. Wilson to be a graduate of that University?

'——— *Street, Jan.* 19, 1818.

'My Dear Sir,—You will be surprised at your letter of the 7th remaining so long unanswered; but the truth is, that I have been confined to bed, and unable to write. I have consulted all the professors whom Dr. Wilson has mentioned in his advertisement, and I have their authority to say, that they *never heard of him or his medicine.* This intelligence you may convey to your correspondent when you please, on my authority.

'I am, my dear Sir, yours truly,

———————

'*To William M———, Esq.* ——— *Square.*'

The following extracts, in parallel columns, one from the work on Medicinal Water, published in 1813, the other from

* Observations on Gout and Rheumatism, p. 28.

that of the Tincture in 1817, shew what veracity is due to this shameless adventurer. The same cases, it will be remarked, are given in both books, with slight variation.

"Abridged cases of cures of gout and rheumatism, 'as convincing proofs of the remarkable efficacy' of

"The Medicinal Water,
Extracted from Dr. Wilson's pamphlet, entitled 'Observations on the Medicinal Water,' published in May, 1813, nearly in his own words, and two years after he had discovered the Tincture!

"Mr. Wright, of Yoxford, (page 17) aged 27, was attacked September 21, 1812, with gout in the great toe, which increasing in severity spread rapidly over the foot.—September 23. All the symptoms of gout have totally disappeared by taking three-quarters of a bottle of the *Medicinal Water*.

"Mr. Salmon, of Glenham, (page 18), aged 60, was seized, early in the morning of January 21, 1813, in the first joint of the great toe and upper part of the foot: administered one drachm and half of Medicinal Water.—January 23. Directed a second dose of the medicine.—January 24. The inflammatory and febrile symptoms are now entirely gone. The patient complains *only* of stiffness in the joints, and slight *tenderness* in walking.

"April 2, 1813. Was again attacked in the left foot.—April 4. The pain was most excruciating. Took two drachms of the *Medicinal Water*; in *eleven hours* after taking the medicine,

"The Tincture,
Extracted from Dr. Wilson's Observations on Gout, &c. published in 1817, nearly in his own words.

"Mr. Wright, of Yoxford (page 138), aged 34, was seized with a violent fit of the gout in April, 1812. By the use of one bottle of the *Tincture* taken at bed-time, the patient in less than forty-eight hours was perfectly free from the gout.

"In the *autumn* of the same year (1812, aged 34) after an exposure to wet and cold, he experienced *another attack equally severe* as the former, and took a similar dose of the *Tincture*, which completely subdued the paroxysm in twenty-four hours.

"Mr. Salmon, at Glenham, (page 140), aged 64, January, 1813, was violently attacked in the left foot. Took half a bottle of the Tincture on going to-bed, and repeated it the following night, which occasioned a gentle perspiration, during the greater part of the night, and after a few hours completely subdued all remains of the disorder.

"April, 1813. Had a *similar recurrence* of gout, and perfectly recovered from the attack in *two* days, by a similar exhibition of the *Tincture*.

the pain and inflammation were entirely gone.

"Mr. Everitt, of Leiston, (page 19), aged 34, January 30, 1813, was seized with gout in both feet and left knee. Took two drachms of the *Medicinal Water*.—January 31. A second dose was taken.—February 1. This morning the patient is free from pain; complains *only* of *weakness* in the joints before affected, and a *slight uneasiness in walking*.

"Mr. Everitt, of Leiston, (page 142), aged 39, January 1813, was violently attacked in both feet and knees. Took half a bottle of the *Tincture* at bed-time, and repeated it the following night. In twelve hours from the time of taking the second dose, the pains, inflammation and febrile symptoms had entirely left him. Mr. Everitt having a strong predisposition to the gout, has *experienced repeated visits of that disorder*, especially in cold or changeable weather, upon all which occasions he has found the happiest effects from the *Tincture*.

"Mr. Folsham, of Yoxford, (page 21), aged 46, complains of severe pains in the larger joints of the upper and lower extremities, which are much aggravated by motion or warmth.—November, 1811, one drachm and half of the *Medicinal Water* were exhibited very late at night.—December 1. The pains in the joints continued severe till one o'clock, when the patient experienced considerable relief.—December 2. Pain, swelling, and redness completely removed from all the joints. This case was thus very successfully completed in about thirty-six hours after the first exhibition of the medicine.

"Mr. Foulsham, of Yoxford, (page 156), aged 48, was the first patient to whom the Tincture was exhibited in attack of acute rheumatism. In November, 1811, Mr. F. was attacked with pains in the larger joints of the upper and lower extremities, greatly aggravated by motion or warmth. I directed him to take two-thirds of a bottle of the *Tincture* at eight o'clock in the evening. The pains in the joints continued with unabated severity till twelve o'clock at night, when they began to abate. In the morning of the second day Mr. F. was perfectly recovered. This case was therefore very successfully completed in about thirty-six hours after the first exhibition of the medicine."

Our readers must have had enough of Dr. Wilson and his Tincture; nor do we think it necessary to go into the proof of what every body knows, that it is, in fact, the Colchicum, as recommended by Mr. Want.

*The Dublin Hospital Reports, and Communications in Medicine and Surgery.* Vol. I. 8vo. Hodges and M'Arthur, Dublin; Cox and Son, London.

*(Concluded from p. 73.)*

*Observations on Hernia;* by CHARLES H. TODD, Member of the Royal College of Surgeons in Ireland, and one of the senior Surgeons to the Richmond Surgical Hospital, House of Industry, Dublin.

Mr. TODD commences this paper with some remarks on the importance of attending to the minute anatomy of the parts implicated with hernia, and particularly of the *fasciæ*. This advice may, perhaps, be very proper in Dublin; but, had the author seen and heard what we have done, he would exclaim *O! jam satis est!*—After an explicit anatomical description of inguinal hernia, as it usually occurs, he mentions an instance in a body brought into the Theatre of the Surgical Hospital, in which he observes—

"On dividing the integuments, the superficial fascia was found unusually thin, with a small quantity of fine cellular substance only between it and the lower and anterior part of the sac; but, on examining the parts connected with the neck of the tumour, I was surprised to find the spermatic cord in an undivided state, extending across the upper part of the sac to its pubal side, and then descending on that side to the posterior surface of the sac, where the testicle was situated In short, this was an instance of direct descent, in which the hernia, instead of passing anterior to the cord, protruded between the cord and the inferior pillar of the ring, so that the cord formed a sort of arch, embracing the neck of the sac for nearly two-thirds of its circumference, close to the external abdominal opening. I am not aware of this peculiar position of the spermatic cord in relation to the neck of the hernial sac, having been described by any author who has written on the subject of hernia; and, until this dissection, I had not conceived the possibility of its occurrence."

But Sir William Blizard, Schmucker, and Le Dran, have related instances of the same kind; and Camper mentions a case in which the spermatic cord was before, and the vas deferens behind, the hernial sac.

Mr. Todd next proceeds to make some remarks on the kind of hernia first described by Mr. Hey, in which the viscus protrudes within the tunica vaginalis of the cord, but covered with another elongation of the peritoneum. This we need hardly observe has since been noticed by Mr. A. Cooper. Mr. Todd does not concur with the opinion generally held respecting the formation of the tunica vaginalis, and consequently objects to the history Mr. Hey has given of the production of the species of hernia to which we have alluded: he says,

"I believe Mr. Hey is not very correct, in stating that in the fœtus a process of the peritoneum is brought down through the ring of the external oblique muscle by the testicle, as it descends into the scrotum. This would lead us to infer, that the tunica vaginalis of the testicle was formed by the mere weight or protrusion of the testicle, in a manner similar to the formation of a common hernial sac. The fact is, that this process of the peritoneum exists in the groin before the testicle has arrived at the ring, and the testicle descends gradually into the scrotum, preserving the same relation to that process as it did to the general sac of the peritoneum while in the abdomen."

We suppose Mr. Todd has deduced this notion from actual anatomical investigation, and we should have felt gratified by an account of the facts which induced him to adopt it; he refers to Bell's Anatomy, but that is by no means satisfactory to us. Mr. Todd then expresses some physiological opinions respecting those parts, "as they appear to him," with which we readily concur; they are such as we were long since taught,—they were the opinions of Hunter. Having mentioned the objection of the author to the generally-received notions respecting this species of hernia, we think it proper to transcribe his own account of it.

"My idea of this form of rupture is, that in it the hernial sac is protruded completely within the cellular sheath of the chord, that, when it descends near to 'the point of insertion of the spermatic vessels into the testicle,' its fundus comes in contact with the upper part of the tunica vaginalis testis, and receives from it, on its lower surface, a serous covering proportioned to the magnitude of the tumor, or the degree of distension of the sac. I am also of opinion that this species of hernia is not peculiar to infancy; on the contrary, I believe it may occur at any period of life, and that it does occur more frequently than surgeons are aware of; at the same time, I am ready to admit, that it must take place more easily in an infant than in an adult, for the following reason. At the time the communication between the peritoneum and vaginal coat of the testicle begins to be obliterated, the testicle has not actually descended into the lower part of the scrotum; it is then rather in the groin than in the scrotum; and even when the obliteration is complete, and for some time after, the two serous membranes are contiguous; it is only as the child advances in life, that the spermatic chord elongates, and a considerable distance intervenes between them: so that, when a rupture takes place in an infant soon after birth, the hernial sac requires to be protruded but a short space from the abdomen, and within the sheath of the spermatic cord, before it comes in contact with the tunica vaginalis testis, to the upper part of which, a moderate degree of distension will cause it to adhere. According as the hernial sac increases in size, its fundus obtains a greater extent of covering from the serous membrane of the testicle, and, when distended, presents a surface, 'smooth and shining as the interior surface of the tunica vaginalis in its natural state,' apparently within that tunic, but actually above it."

In considering the cause of the rising upwards of crural hernia, Mr. Todd mentions the explanation of it given by Mr. Lawrence, page 349 of his Treatise, where he says,

"Since the motion of the thigh and the more close adhesion of the integuments to the subjacent parts resist the increase of the tumour downwards, and the larger quantity of cellular and adipose substance at the bend of the limb offers less resistance, it comes forward to the surface, so as to lie in general in front of the crural arch."

He very justly observes, that

"Mr. Lawrence's explanation appears perfectly satisfactory; but, when the student compares it with Mr. A. Cooper's description of the superficial fascia, he cannot subscribe to the accuracy of both. Mr. Cooper says, 'When the skin is removed from the crural arch and forepart of the thigh, a thin fascia will be seen to extend over the tendon of the external oblique muscle, *and to adhere firmly to the edge of the arch*.'

"Now, were this the case, it must be obvious that, as the superficial fascia forms one of the coverings of the hernial sac, its connexion with the crural arch would oppose the ascent of the tumour, and the increase of the swelling downwards would occur with much greater facility. A careful dissection of these parts, however, shews us that the superficial fascia is really as loosely connected to the lower border of the obliquus externus, as to any of the other subjacent parts, and that, when exposed, we can move it with our fingers on the parts over which it is expanded, with equal ease every where, except at the hollow of the thigh, where the cutaneous vessels and nerves, the lymphatics and their glands, are entangled with its laminæ."

We believe every one that will take the trouble of carefully examining the parts, will acknowledge the accuracy of these observations. In performing the operation for crural hernia, he prefers the division of Gimbernat's ligament directly towards the pubes, without seeming to be aware that this mode of operating is becoming general, we believe, throughout Europe. Practical remarks are made on the treatment of the disease, and on some points of importance in the operation; which, although evidently deduced from original observation, are only conformable to what had previously been published on this subject. So many men of eminent abilities have within these few years past almost wholly devoted themselves to the investigation of the nature of hernia, that the acquisition of farther knowledge respecting it can hardly be expected.

---

*Observations on some important Points in the Practice of Military Surgery, and on the Arrangement and Police of Hospitals; illustrated by Cases and Dissections;* by John Hennen, Deputy Inspector of Hospitals. Edin. 1818. 8vo. pp. 496.

In a preface the author apologizes for omitting to insert many engravings of diseased parts, the drawings for which are in his possession. With much truth he remarks, that the expence of his work would thence have been too much enlarged. He might have added the difficulty of procuring artists sufficiently acquainted with those important minutiæ which form the leading characters of diseases. Drawing has been for some time considered part of a surgeon's education, and we hope to find etching become so likewise.

The introductory remarks contain an historical account of military surgeons, from the time that the army can be said to have had any medical establishment: in the early periods, almost indeed to our own times.

"A very few years have elapsed since Military Surgery was at so low an ebb in England, that one of the most able and enthusiastic medical philosophers which the country ever produced, has made the following observations on the subject. 'Practice, not precept, seemed to be the guide of all who studied in this branch; and, if we observe the practice hitherto pursued, we shall find it very confined, being hardly reduced to the common rules of surgery, and therefore it was hardly necessary for a man to be a surgeon to practise in the army.' This opinion of Mr. Hunter, who was himself an army surgeon, little as it flatters his predecessors, was, to a great extent, founded on truth; but, if we come to investigate the cause of this deficiency in practice, and this scantiness of precept, we shall be able easily to trace it to one of the most powerful springs of action implanted in the human mind. He must have been indeed possessed of a most glowing enthusiasm, and an utter contempt for self-interest, who would have buried his talents and his industry in a situation where obscurity, poverty, and neglect, spread all their horrors before him. These were, for years, the portion of the army-surgeons; their situation was looked upon as the lowest step of professional drudgery and degradation; and, if a man of superior merit by chance sprung up in it, or entered it for temporary purposes, he soon abandoned the employment for the more lucrative, the more respectable, and the less servile, walk of private practice. The school was good; but the best and most natural feelings of the human heart were too deeply lacerated, to permit an independent man to continue long a pupil."

The truth is, that the establishment of the French Academy of Surgery should be considered the æra at which surgery first rose to the rank of a science. As the term expresses it, the *Chirurgeon* was only to use his hands in the manner the physician directed him. Though we borrow the term from the Greek language, the distinct office was unknown among those people. Among the Latins it was rarely known, and, as his office was confined to operations, practice was all that he required. It is universally known that, in England, surgeons were not incorporated as a body till the time of Henry the Eighth, when they chartered under the title of Barber-Surgeons. This continued to so late a period that the race of Barber-Surgeons have not been extinct much more than twenty years. In their number were the celebrated Pott and his colleagues at St. Bartholomew's. We suspect Grindal was the last.

Mr. Hennen continues his historical account through other parts of Europe: when returning home, he remarks,—

"In England, within the period of the French Revolution, few practical surgeons have written exclusively on military surgery. One towering genius has touched upon it, as a part of that immortal monument which he has raised to his own fame, and to the glory of the British surgical name, in the 'Treatise on the Blood, Inflammation, and Gunshot Wounds.' This great work of Mr. John Hunter was published in 1794, after his death. The materials were collected during a period of active service abroad, whither he went as senior staff-surgeon in the year 1760, and served at Bellisle and in Portugal for the remainder of the war. In the year 1790, he was appointed to the joint offices of surgeon-general to the army, and inspector of regimental infirmaries; and in conjunction with the physician-general, Sir Clifton Wintringham, held the superintendence of the department. Staff-surgeons, as well as physicians, were, at this period, and for many years before, selected from civil life on the spur of any expedition, and very rarely from the regimental surgeons. The names of Pringle, Brocklesby, and Monro, survive in their works; but, previous to the time of Mr. Hunter, no publication with which I am acquainted was produced by the staff or regimental surgeons; although the appointment of the latter was coeval with their corps, and many of them had consequently, ample opportunities for observation. On the lamented death of Mr. Hunter, a board was formed, which dates from his present Majesty's order, issued through the secretary-at-war, in October 1793. This consisted of a physician-general, a surgeon-general, and an inspector of regimental infirmaries, who were invested with distinct but jarring powers, and continued to act until 1808, when it was dissolved, and new modelled, upon the basis of responsibility as a body, which was constituted of a director-general and two principal inspectors. On the peace establishment of 1817, the two latter officers ceased to be borne on the returns of the staff of the army, but, in the army-list of January 1818, the appointment of a deputy-director-general appears.

"The circumstances of a long war gave rise to many divisions of rank among the officers of the medical staff; but several of these were abolished by the King's warrant in 1804; and the staff is now composed of a certain number of inspectors and deputies, physicians, surgeons, apothecaries, assistant-surgeons, and hospital-assistants, with commissions, and of some hospital mates and dispensers, who hold their situations by warrant."

The observations contained in this work, we are informed, are the results of an active military life, since the first year of the present century, in the Mediterranean; throughout the whole of the Peninsular campaigns; in the Hospitals in France; and during the late short but bloody campaign on the continent. Mr. Hennen says,

"The medical philanthropist will naturally ask, what results have accrued from such ample sources of experience? What progress has been made in softening the miseries of pain and disease, and in extracting from such multitudes of victims, antidotes to the waste of human life? The young practitioner also, who may enter the service of his country, will enquire, where am I to collect the fruit of that experience, with which so many campaigns have enriched my predecessors? and how, if the opportunities come within my reach, am I best to avail myself of them? It is in some degree to answer these interrogatories, that I have ventured to make the following observations."

We may remark that it was in the same school that Paré, Petard, and Wiseman, were educated, and in which Hunter collected many of those facts, and commenced those investigations, which were eventually to disclose to us all that, probably, we shall ever know of the functions of the human body; and in the list of those who, from the ravages of war, have deduced the means of benefit to mankind, we must not omit to place the name of Baron Larrey. Several pages then follow containing observations on the conduct army-surgeons should adopt in the exercise of their professional duties and conformity to military discipline; and the preparatory measures advisable previously to taking the field. We pass over this to the part of the work more interesting to general readers, which commences with the consideration of the "*General nature and first treatment of wounds, course of balls, &c.*"

We may date from the time of Paré the commencement of rational notions respecting the nature of Gun-shot wounds.

The ideas of the "burnings" and "poisonings" attending them are now justly exploded; they are merely considered as lacerated and contused wounds, varying according to the nature of the parts injured, and the degree of velocity with which the separation may have been effected.

After noticing the slight degree of pain and hæmorrhage attending them, when no large nerves or blood-vessels are injured, the various effects produced in different men are described.

"Some men will have a limb carried off, or shattered to pieces, by a cannon ball, without exhibiting the slightest symptoms of mental or corporeal agitation; nay, even without being conscious of the occurrence; and when they are, they will coolly argue on the probable result of the injury: while a deadly paleness, instant vomiting, profuse perspiration, and universal tremor, will seize another on the receipt of a slight flesh wound. This tremor, which has been so much talked of, and which to an inexperienced eye is really terrifying, is soon relieved by a mouthful of wine or spirits, or by an opiate; but, above all, by the tenderness and sympathizing manner of the surgeon, and his assurances of his patient's safety."—"The ball will frequently have passed nearly through the limb, and be retained only by the elasticity of the common integuments. There we cut upon, and extract it at once; and we should lay it down, as a rule not to be deviated from, to extract on the spot every extraneous body that we possibly can, either by the forceps alone, or with the aid of a bistoury."

Some remarks ensue on the devious track of balls; and it is observed that, when the blush or weal in the skin, as noticed by Mr. Hunter, is not witnessed, a certain emphysematous crackling discovers its course, and leads to its detection.

"The ball is, in many instances, found very close to its point of entrance, having nearly completed the circuit of the body. In a case which occurred to a friend of mine in the Mediterranean, the ball, which struck about the Pomum Adami, was found lying in the very orifice of its entrance, having gone completely round the neck, and being prevented from passing out by the elasticity and toughness of the skin, which confined it to this circular course. This circuitous route is a very frequent occurrence, particularly when balls strike the ribs, or abdominal muscles; for they are turned from the direct line by a very slight resistance indeed, although they will run along a continued surface, as the length of a bone, along a muscle, or a fascia, to a very extraordinary distance at times. If there is nothing to check its course, and if its momentum is very great, it is surprising what a variety of parts may be injured by a musket-ball. I have seen cases where it has traversed almost the whole extent of the body and extremities. In one instance, which occurred in a soldier, with his arm extended, in the act of endeavouring to climb up a scaling ladder, a ball, which entered about the centre of the humerus, passed along it, over the posterior part of the thorax, coursed along the abdominal muscles, dipped deep through the glutæi, and presented on the fore-part of the opposite thigh, about midway down. In another, a ball, which struck the breast, lodged in the scrotum, the man standing erect in the ranks."

"I was first led to an examination of the passage of balls, not only along a convex surface, but also along a concave, from seeing the course of some musket balls, which had deeply grazed along, but not penetrated, the arm, when it was in a curved position, as in a soldier when firing his musket. In this posture of the soldier, I have frequently seen the mark of the ball commencing at the wrist, and, instead of going through, or perhaps flying off at a tangent, and striking the breast, go all round between the shirt and skin, furrowing the latter, and going out at the point of the shoulder; thus describing a portion of the circumference of a circle."

Mr. Hennen next proceeds to make some remarks on the field-treatment of the more complicated cases arising from gun-shot wounds.

"It frequently happens that an arm or leg, or perhaps both, are carried completely off by round shot, leaving an irregular surface of jagged and lacerated soft parts, and a projecting bone shivered to pieces. The obvious plan to be followed in this case, is to reduce this horrid-looking wound to the simple state of a limb which has been separated by art."

"It not unfrequently occurs that the arm is carried clean out of the socket; and in this case very little more remains for the surgeon to do than to pass a ligature round the arteries, *even though they do not bleed, as often happens*,—to cut short the lash of nerves, which, in this case, usually hangs far out of the wound,—to bring the lips towards each other by adhesive straps, and to support them by proper compress and bandage."

Excision of the head of the humerus is not considered under any circumstances proper as a *field*-operation.

"Extensive injuries of the joints form an urgent class of cases for immediate amputation. I am well aware that some very favourable joint-cases have ended successfully without removing the limb; but I will venture to assert, that the pain and inconvenience of the cure, the inability of the member, and its proneness to disease, have infinitely counterbalanced the benefit derived from saving it."

"In civil society, where the patient has always led a temperate quiet life, and the injury has been inflicted perhaps by a clean cutting instrument, or a small ball has passed near, or partially injured the joint (a case so very different from that occasioned by a large shot passing into or near a joint, where the patient has to be dragged over heavy roads, in bad carriages, without surgical aid or medical comforts), I know that cures have been effected; and, even in military life, there are instances to be found of the same kind. I would still, however, lay it down as a law of military surgery, that no lacerated joint, particularly the knee, ankle, or elbow, should ever leave the field unamputated, where the patient is not obviously sinking, and consequently where certain death would follow the operation.

"3dly. Under the same law are included, by the best and most experienced army-surgeons, all compound fractures close to the joints, especially if conjoined with lacerated vessels or nerves, or much comminution of the bone, particularly if the femur is the injured bone.

"4thly. Extensive loss of substance, or disorganization of the soft parts, by round-shot, leaving no hope of the circulation being carried on, in consequence of torn arteries or nerves.

"5thly. Cases where the bones have been fractured or dislocated, without rupture of the skin or great loss of parts, but with great injury or disorganization of the ligaments, &c., and injuries of the vessels, followed by extensive internal effusions of blood among the soft parts."

The question respecting the propriety of immediate amputation in cases in which it will eventually be necessary, is next considered. The opinion of Mr. Hennen is decidedly in favour of it, and such appears to be the case with military and naval surgeons in general.

Mr. Hennen says, "Mr. Hunter is the leading English writer who has thrown any doubts upon the question; but he considered it theoretically." We cannot admit this—Mr. Hunter did not want experience in injuries of this kind, and he promulgated no opinions which were not deduced from the consideration of facts. We must, however, allow, that the opportunities he had for observation were under partial circumstances.

In advising immediate amputation, the author observes,

"While hundreds are waiting for the decision of the surgeon, he will never be at a loss to select individuals who can safely and advantageously bear to be operated on, as quickly as himself and his assistants can offer their aid: but he will betray a miserable want of science indeed, if, in this crowd of sufferers, he indiscriminately amputates the weak, the terrified, the sinking, and the determined. While he is giving his aid to a few of the latter class, encouragement and a cordial will soon make a change in the state of the weakly or terrified; and a longer period and more active measures will render even the sinking proper objects for operation. If, however, he is disappointed in his hopes, surely the dictates of common sense will point out the necessity of procrastination, and not induce the surgeon to perform what he knows must ultimately be done, at a period where it is manifestly counteracting the object he has in view to do itat once."

In cases of compound fracture, particularly of the lower extremities, little more is advised to be done on the field than the removal of splinters and extraneous bodies that can be easily accomplished; and to place the limb in the most easy posture, until the patient arrives at his place of final destination. A caution is given on the necessity of tying any large artery that may be injured, when it can be got at, although it may not bleed.

The proper arrangement of the receiving hospital, and the duties of the medical attendants, are pourtrayed in a manner that shows the attention the author has paid to this important subject. This part of the work contains much interesting information for the military surgeon.

The "*Dressings and general Medical Treatment*" are next considered.

After some observations on the local treatment of simple wounds, the author deprecates the prejudice that exists among some of the younger surgeons, on the subject of phlebotomy, as applied to soldiers; supposing they cannot bear the loss of blood so well as the lower order of persons in civil life; but the reverse is really the case.

This *prejudice*, and its consequences, will be lamented as long as men are disposed to consider, that, what is termed *systematic knowledge*, can render the more toilsome means of studying facts in their series and order unnecessary for enabling them to exercise the duties of their profession. But it was not thus that Sydenham, Jackson, and some others whom we have so often had occasion to mention, acquired that which will bear their names to posterity amongst those of the benefactors of human nature.

"Few, if any, (the author observes,) of the veterans are without confirmed hepatic affections, or a strong tendency that way." This arises from their irregular mode of life, and the great propensity they have for drinking spirits. Much mischief is also often done them by the mistaken kindness of their comrades in the hospitals, or in their journey to them, which gorges the wounded with food and intoxicating liquors. Much care is necessary to prevent the formation of sinuses, and, if pressure be not effectual for this purpose, the free use of the knife will be advisable. He very properly dwells on that important part of surgical manipulation, the application of a bandage, and says,

"The most judicious medical treatment and the ablest surgical operation will fail, if not assisted by good bandaging; and errors in both will soon be recovered, if a proper system is adopted. I have seen innumerable instances of most promising stumps degenerating in a few days under an inefficient or careless dresser; and I have even traced some deaths to such a cause; while rapid amendment and the saving of a limb often result from the due use of a proper system of dressing and applying the roller."

The ensuing section is on *the extraction of foreign bodies.* This is a subject which does not afford an opportunity of evincing much novelty of observation. Some cases are adduced, to shew the magnitude of extraneous bodies that may lie embedded in muscular parts, without being detected, until at length inflammation is excited by them; pointing out their situation. There are also some curious instances of the insertion of foreign bodies into wounds, from the persons of those who may have stood near their patient. We transcribe the following—

"A Hanoverian soldier received a severe wound from a grape-shot on the 18th of June 1815, at Waterloo, which struck him on the external part of the thigh, producing very extensive laceration. On the second day he was brought into the hospital, and the usual dressings applied. On the fifth day a long narrow passage was discovered by the probe, seeming to run nearly the whole length of the vastus externus muscle. On cutting into this, three pieces of coin (which, from the very curious mode in which they were compacted together, I thought worthy of presenting to the director-general of hospitals) were extracted from the parts. This poor fellow, a raw recruit, had no money whatever about him, nor even a pocket to contain it in, and fervently protested against his right to this forced loan. He accounted for it by supposing it carried from the pocket of his comrade, who stood before him in the ranks, and who was killed by the same shot.

"I cannot omit noticing here a trait strongly illustrative of the mobility of mind which characterizes soldiers, and their proneness to superstition and belief in omens, which a surgeon acquainted with their character can often turn to their benefit. The part of those two coins which had been flattened out happened to be that on which Napoleon's head was impressed. From one it was nearly effaced; and on observing this circumstance to the patient and his comrades, an universal burst of joy echoed through the ward; the young Hanoverian exulted in the share he conceived he had personally had of contributing to the downfall of the French Emperor. His health rapidly improved, and I have no doubt that this simple circumstance had a good effect upon every man who witnessed it."

Very serious effects frequently ensue from *contusions from shot and shell.* Mr. Hennen observes—

"It very often happens that, while all is smooth and sound to the eye, or there is perhaps only a slight erosion of the skin, a very serious injury has been done to the subjacent parts. This is more particularly the case where a spent ball of large size grazes along any of the cavities; or where they have received a severe injury from the running over of a gun, or the explosion of an ammunition waggon, or other violence; on all these occasions, great advantage will be derived from taking a few ounces of blood from the arm, and embrocating the contused part with some linimentum saponis, or any other mild stimulant."

When the vitality of the part is entirely destroyed, tedious sloughing and ulceration is the consequence, or a chain of ill-conditioned abscesses form, burrowing deep beneath the disorganized mass of skin and integuments, if not prevented by timely evacuation. The effects of severe blows by spent or oblique shots striking the head, thorax, and abdomen, are still more dangerous.

"Under this class is to be ranged death from the wind of a ball, which has given rise to such a multiplicity of fables, and on which so much argument has been exhausted. I should be very far from denying altogether the influence of the shock, whether that is electrical or not; because we frequently meet with cases where no local injury can be detected after death. That the compressed air alone, or the friction of the ball, has no such effect, appears to me satisfactorily proved by the usual arguments, drawn from instances of near comrades being killed, or parts of the body torn off without the individual being destroyed; and it is rendered, if possible, still stronger by instances of escape, owing to a sudden contortion of the body, in evasion of the summary military punishments inflicted in some foreign countries, by blowing men off from the mouth of a gun."

Observations on *injuries of the bones*, occupy the next section,—a class of accidents, which, from the immediate and eventual effects they induce, are of the most serious importance. In ascertaining the nature of the injury received, Mr. Hennen observes, that, some information may be derived from the appearance of a ball after being extracted from a wound. When it has brushed obliquely by a bone, and injured its external plate, its surface is often jagged, and presents the appearance of a file clogged with raspings of ivory. Balls most frequently lodge in and perforate the heads of bones: in these cases the injury is comparatively simple in the recent state; but they almost constantly turn out cases for secondary amputation. When a ball has lain long in a bone, the cancelli break down and admit of its rolling about in the cavity; sometimes they remain without inconvenience for several years; but in the greater number of cases produce such effects as to render amputation of the limb necessary.

The treatment of *compound fractures* is next considered. In those of the thigh Mr. Hennen gives the preference to the straight position of the limb—

"I am warranted, from most ample experience, to infer, that lying on the back with the limb extended, is by far the most tolerable to the patient, and admits of much easier access and dressing; and, what is still more important, is, in its ultimate success, equal, if not superior, to either the bent position of Pott—the patient on his side, or the semiflexion of the knee—the patient on his back, and the limb in a fracture box."

Some observations follow on the sufferings and danger a wounded soldier undergoes in his removal to the hospital; on the propriety of deferring any attempt at accurate coaptation of the broken bones until he has arrived there, and the inflammation has subsided; and on the treatment of wounds, and diseases of the bones which accompany, or are consequent on, this species of injury. The extensive experience of Mr. Hennen has furnished him with an ample series of facts, and his judicious reflections on them render this part of the work particularly interesting. We cannot attempt, within our restricted limits, to convey to our readers an idea of the information it contains; and we will not do the author so much injustice as to make choice of any passage, where all is so deserving of the serious attention, both of military and civil practitioners. We may, however, transcribe the following account of a peculiar species of injury, because it has not been particularly adverted to by any author.

"I shall now advert to a species of the comminuted compound gunshot fracture, which, although at first of but little consequence in appearance, is of most serious importance in its results. This occurs where a musket-ball has perforated a cylindrical bone, without totally destroying its continuity, and, consequently, without producing any distortion of the limb, or other symptoms which characterize a fracture. The foundation of infinite mischief is, however, laid, for not only is the shaft of the bone injured, but fragments are carried into and lodged in the medullary canal; and, if the limb has been in an oblique position, or that the ball has taken an oblique course, these fragments are often driven in to a great distance, and firmly impacted in its cavity, there keeping up a constant and uncontrolable irritation, and destroying both the medulla and its membrane, together with the cancelli, which naturally support it. I have repeatedly seen this separated portion of bone, lying in the medullary canal, at the distance of from four lines to an inch and half from the circular hole formed by the passage of the ball, retaining its shape and its solidity, while all the surrounding osseous parts were diseased, and formed a spongy dis coloured mass of bony granulations around it; the periosteum for some way, both above and below the wound, being entirely separated from the bone. To attempt to save such a limb, is imposing a task on the powers of nature, which nineteen times in twenty she is unable to effect, even under the most favourable circumstances."

The ensuing section is on *injuries of the joints.* These are cases which most frequently require immediate amputation. Mr. Hennen says he has met with only two, where the limb has been saved after serious injury of this kind. The disposition of soldiers to copious drinking of spirituous liquors, and the distance they are frequently carried over rough roads, to the hospital, add considerably to the injury. Mr. Hunter mentions the case of a man who was shot through the joint of the knee: the ball entered at the outer edge of the patella, crossed through the joint under that bone, and came out through the inner condyle of the os femoris. This man had nothing done to his wounds for four days after receiving them, having secreted himself in a farm-house: when brought to the hospital the wounds were only dressed superficially, and he got well.

A very interesting case is related of Lieutenant-colonel R. who received a musket-shot on the outside of the knee-joint. The course of the ball was never ascertained, but a portion of the patella was broken off, and found on the opposite side of the joint. Circumstances prevented the immediate removal of the limb, which was the intention of the attending surgeon. On the third day Mr. Hennen saw him: inflammation was then about to take place, and there was a considerable degree of tumefaction of the whole limb. Copious abstraction of blood, generally, and by means of leeches, and the most rigorous abstinence in diet, were had recourse to. Valsalva's plan for the cure of aneurism was excess to it. About 250 ounces of blood were lost within seven days. These measures were successful. Something may be attributed to the patient's state of body at the time of receiving the injury, of which we shall transcribe the account given by the Colonel himself to Mr. Hennen.

"Owing to circumstances of the service on the 16th June 1815, I had a common tea breakfast, and at night, after a fifty-miles march, a piece of bread, with a little spirits and beer. On the 17th, I had a meat breakfast, and, throughout the day, was employed in a very severe skirmish in heavy rain. At night I took a small piece of bread, and a little spirits. On the 18th, I took for breakfast, at seven o'clock in the morning, a very small quantity of meat, and one glass of wine.

"*Sunday,* 18*th June, Waterloo.*—About two o'clock, I received a musket-shot in the outside part of the right knee-joint; a surgeon, who saw it almost immediately, was prevented cutting out what was then thought to be the ball, protruding on the opposite side of the knee-pan, by the heavy fire of the enemy. I moved back towards the village of Waterloo, and on the road met with another surgeon, who looked at my wound, and it was decided that amputation above the joint was the only means of saving my life. The instruments were brought for the purpose, when a reiterated attack by the enemy's cuirassiers caused orders to be issued for our immediate removal. I moved on to Brussels, where I arrived at half past eight P. M. I had my limb washed, was stripped and put to bed. No dressing or application whatever was used, but I received a caution from a medical gentleman, who accidentally saw me, to take only lemonade; my diet, therefore, this day, was water and lemonade."

When an attempt to save a limb is thought advisable, a variety of circumstances must be taken into consideration: besides the nature of the injury, constitution, &c. of the patient, the possibility of his enjoying rest and the facilities afforded to his medical attendants of seeing him and enforcing their orders must be maturely weighed.

A frequent and serious consequence of gun-shot wounds is *contracted extremities*, originating from loss of parts, or rigidity of the joints, from the effects of inflammation. Mechanical measures are recommended for restoring the natural state of the limbs, as bandages, splints, and juga of different kinds.

*(To be concluded in our next.)*

---

